# On Poisons in Relation to Medical Jurisprudence and Medicine

**Published:** 1876-01

**Authors:** 


On Poisons in Relation to Medical Jurisprudence and Medicine. By Alfred Swaine Taylor, M.D., F.R.S., Fellow of the Royal College of Physicians, and Lecturer on Medical Jurisprudence in Guy's Hospital. Third American, from the third and thoroughly revised English edition. With 104 illustrations. Philadelphia: Henry C. Lea. 1875.

This valuable work, which for so many years has been the accepted authority, both in England and the United States, on medical jurisprudence, needs no word of commendation at our hands. The whole work has been remodeled, and in rewriting many of the chapters the author has availed himself of the recent advances in the collateral sciences of chemistry and pathology. As a manual for the use of students and practitioners of law and medicine, there is no better work. No medical or law library is complete without it.

